# Moderate and transient impact of antibiotic use on the gut microbiota in a rural Vietnamese cohort

**DOI:** 10.1038/s41598-022-24488-9

**Published:** 2022-11-23

**Authors:** Vu Thi Ngoc Bich, Ngoc Giang Le, David Barnett, Jiyang Chan, Niels van Best, Tran Dac Tien, Nguyen Thi Hien Anh, Tran Huy Hoang, H. Rogier van Doorn, Heiman F. L. Wertheim, John Penders

**Affiliations:** 1 Oxford University Clinical Research Unit, Hanoi, Vietnam; 2 School of Nutrition and Translational Research in Metabolism, Department of Medical Microbiology, Infectious Diseases and Infection Prevention, Maastricht University Medical Center+, Maastricht, The Netherlands; 3 Maastricht Centre for Systems Biology (MaCSBio), Maastricht University, Maastricht, The Netherlands; 4 Center for Disease Control and Prevention, Ha Nam, Vietnam; 5 National Institute of Hygiene and Epidemiology, Hanoi, Vietnam; 6 Centre for Tropical Medicine and Global Health, Nuffield Department of Medicine, University of Oxford, Oxford, UK; 7 Department of Medical Microbiology and Radboudumc Center for Infectious Diseases, Radboud University Medical Center, Nijmegen, The Netherlands; 8 CAPHRI Care and Public Health Research Institute, Department of Medical Microbiology, Infectious Diseases and Infection Prevention, Maastricht University Medical Center+, Maastricht, The Netherlands; 9 Institute of Medical Microbiology, RWTH University Hospital Aachen, RWTH University Aachen, Aachen, Germany

**Keywords:** Microbiology, Molecular biology, Gastroenterology

## Abstract

The human gut microbiota has been shown to be significantly perturbed by antibiotic use, while recovering to the pre-treatment state several weeks after short antibiotic exposure. The effects of antibiotics on the gut microbiota have however been mainly documented in high-income settings with lower levels of antibiotic resistance as compared to lower and middle income countries (LMIC). This study aimed to examine the long-term consequences of repeated exposure to commonly use antibiotics on the fecal microbiota of residents living in a low income setting with high prevalence of antibiotic resistance. Fecal samples from household individuals (n = 63) participating in a rural cohort in northern Vietnam were collected monthly for a period of 6 months. Using 16S V4 rRNA gene region amplicon sequencing and linear mixed-effects models analysis, we observed only a minor and transient effect of antibiotics on the microbial richness (ß = − 31.3, 95%CI = − 55.3, − 7.3, *p* = 0.011), while the microbial diversity was even less affected (ß = − 0.298, 95%CI − 0.686, 0.090, *p* = 0.132). Principal Component Analyses (PCA) did not reveal separation of samples into distinct microbiota-based clusters by antibiotics use, suggesting the microbiota composition was not affected by the antibiotics commonly used in this population. Additionally, the fecal microbial diversity of the subjects in our study cohort was lower when compared to that of healthy Dutch adults (median 3.95 (IQR 3.72–4.13) vs median 3.69 (IQR3.31–4.11), *p* = 0.028, despite the higher dietary fiber content in the Vietnamese as compared to western diet. Our findings support the hypothesis that frequent antibiotic exposure may push the microbiota to a different steady state that is less diverse but more resilient to disruption by subsequent antibiotic use.

The crucial importance of the human gut microbiota in modulation of host health, including prevention of infections, immune-mediated, metabolic and neurological diseases, has been well-established^1,2^.

After the initial maturation of the gut microbiota during the first years of life, it exists as a highly diverse and relatively stable ecosystem in healthy individuals^3,4^. Transient or long term alterations can however still occur in response to various external factors such as diet, living conditions and medical drug use^5^. In general, the human gut microbiota is rather resilient and recovers after drastic pulse perturbations, such as courses of antibiotics. Maintaining a resilient and robust microbiome is pivotal to minimize the period of microbial disruption upon antibiotic use which may put the host at increased risk of opportunistic infections^6^.

Microbiota resilience after antibiotic exposure has been shown to be highly dependent on diet, environmental context, past perturbations, and specific properties of the used antibiotics, as well as the individual-specific microbiome^7,8^. Regarding the latter, four main factors have been recognized to play an essential role in the resilience and recovery of the human gut microbiota: initial composition, capability to maintain its functional redundancy, abundance of (opportunistic) pathogens and the antibiotic resistance genes (ARGs) reservoir^9,10^. Indeed, when a simulated human gut microbiota was exposed to amoxicillin, it was shown that its biodiversity was extended by increasing abundance of pathogens carrying resistance genes^11^. A study on 12 healthy adults found that bacterial species harboring β-lactam (ARGs) modulated the resilience/recovery patterns by increasing the chances of survival and novel colonization after a broad-spectrum antibiotic treatment^12^.

β-lactam antibiotic therapy has been associated with profound alterations in the gut microbiota of both healthy individuals and patients, including a significant decrease in species richness, changes in abundance of specific bacterial taxa and a relative increase in antimicrobial resistance^13^. For instance, previous studies demonstrated that the abundance of Enterobacteriaceae was decreased after treatment with penicillin and cephalosporin antibiotics while the abundance of enterococci and *Bacteroides* spp was increased^5,14^. Moreover, the ensuing perturbations related to the use of β-lactam antibiotics is conductive for the selective outgrowth of opportunistic pathogens as illustrated by the increased risk of *Clostridioides difficile* infection^15,16^. A recent study illustrated that a course of penicillin or amoxicillin does not alter human gut microbiota of western obese individual^17^, whereas other studies found more profound yet temporary microbiota alterations upon amoxicillin treatment^18^. Previous culture-based studies reported that bacterial abundance returned to pre-antibiotics state 4 to 14 days after discontinuation of cephalosporin use^19–21^, but some studies observed an altered state that lasted longer than 42 days^22,23^.

The majority of studies were conducted in high-income settings or settings with a low prevalence of antibiotic resistance. Studies on impact of antibiotics on microbiota conducted in low- and middle-income countries (LMICs) with a high consumption of antibiotics and high prevalence of antibiotic resistance are underrepresented^24^. The effects of antibiotics on the microbiota composition in these settings may differ given the different initial microbiota composition resulting from different lifestyles, dietary habits and history of antibiotic exposures. The fecal microbiota composition of rural populations in LMICs like Vietnam, is expected to be more diverse than in high income countries^25^ at least in part due to the lower fat and higher fibre-content of the diet^26,27^ as compared to the Western diets which are typically enriched in saturated fats and simple carbohydrates^28,29^. We therefore conducted this study (i) to explore the long-term impact of antibiotics on diversity of the human gut microbiota in a resource limited setting and (ii) to evaluate whether repeated exposure to commonly used antibiotics induces the human gut microbiota with high prevalence of antibiotic resistance to be resilient to further antibiotic effects.

## Study design and methods

### Study cohort, study design and fecal sample collection

This study was conducted within the context of an existing prospective household-based community cohort in Ha Nam in northern Vietnam, which was established to quantify the burden of influenza and to gain insights into the influenza virus transmission in a tropical setting. A full description of the demographics and lifestyle of the Ha Nam cohort has been published previously^30^.

To examine the impact of antibiotic use on shifts in the gut microbiota of residents of this community, we conducted a longitudinal study over a period of six months from November 2014 to June 2015. The study design encompassed weekly interviews to collect information on health issues and consumption of antibiotic use in the preceding week and monthly fecal samples for microbial profiling. The interviews included questions to indicate the indications for which antibiotics were used in the preceding week (including having respiratory symptoms (cough, breathless, sore throat)).

From 80 households (HHs), we selected 11 HHs in which at least one household member used antibiotics during the six months follow-up. In total this resulted in 41 participants of whom 32 used at least one course of antibiotics.

In addition, we randomly selected a control group from 11 HHs (22 participants) where none of the household members used antibiotics during the study period. We used randomizer (www.randomizer.org) for the selection.

**Figure 1:**
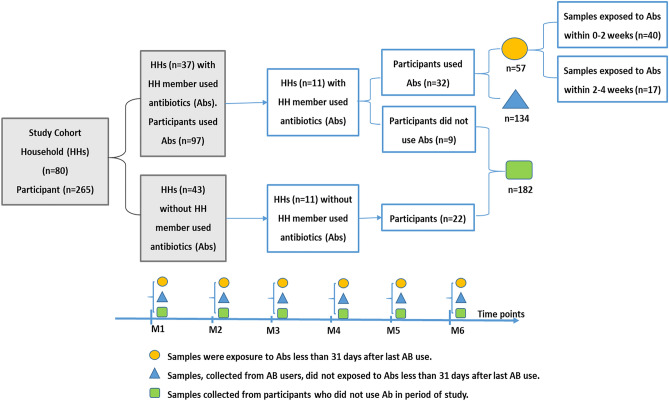
Study design and classification of samples into categories based on the number of days of exposure to antibiotics prior to sampling. The blue boxes indicate HHs, subjects and samples are part of the assessment. The light grey boxes indicate HHs of the study cohort and part of participants whose samples did not be included in the assessment.

To investigate the effects of antibiotic use, samples were divided into one of three categories: samples collected within two weeks after last antibiotic use (E_2_), samples collected within two to four weeks after last antibiotic exposure (E_4_), and samples collected without any antibiotic exposure in the preceding four weeks (Fig. 1).

To compare the microbial diversity between our cohort and a cohort with less antibiotic use, we included a random selection of healthy Dutch adults (≥ 18 years) who participated in the Carriage Of Multiresistant Bacteria After Travel (COMBAT) Study. This study was conducted to evaluate the travel-associated risk factors on the acquisition, persistence and transmission of antimicrobial resistance (AMR) in gut microbiota of healthy people. The detailed information of demographic characterization of the cohort has been reported previously^31^. For the purpose of the present study, we only included fecal samples collected at baseline (before travel) of 106 subjects with no history of antibiotic use during the past year. Differences in Shannon diversity and observed species richness in fecal samples of Dutch and Vietnamese individuals were examined by linear regression analyses with adjustment for age and sex as potential confounding factors.

We used the term “microbial resistance to pulse perturbations” to indicate the resistance of the microbiota to change upon exposure to antibiotic use. Microbial resilience, on the other hand, reflects the recovery speed upon a shift in the microbiota. In this study, to examine the microbial resistance to pulse perturbations, we stratified Vietnamese individuals who used antibiotics according to their microbial resistance. For this purpose, we calculated the Aitchison distance between the samples of each subject collected before and immediately after antibiotic use. Subjects were classified as having a low microbial resistance when the Aitchison distance was above the median, whereas subjects with an Aitchison distance below the median were classified as having a high microbial resistance to pulse perturbations changes.

We also examined if the number of different antimicrobial resistance genes (ARGs) encoding extended-spectrum beta-lactamases, carbapenemases and plasmid-mediated colistin resistance in fecal samples was associated with the microbial community structure.

### DNA extraction, 16S –rRNA amplicon sequencing and determination of antimicrobial resistance genes

Fecal samples were transported within 4 h after defecation to the National Institute of Hygiene and Epidemiology (NIHE) in cold conditions (4–8 °C). Within 3 h after transport, samples were stored at − 80 °C until DNA extraction. Microbial DNA was manually extracted from frozen feces according to protocol Q of the International Human Microbiome Standards consortium^32^. Briefly, fecal samples were firstly subjected to mechanical lysis by repeated Bead-Beating (RBB) followed by column-based purification.

We performed amplicon libraries preparation and sequencing as described previously^33^. In brief, we amplified the V4 region of the 16S rRNA gene from each DNA sample in triplicate using the 515 f./806r primer pair^34^. PCR amplicons of the triplicate reactions were pooled; next products were purified using AMPure XP purification (Agencourt, Massachusetts, USA) according to the manufacturer’s instructions and eluted in 25 µl 1 × low TE (10 mM Tris–HCl, 0.1 mM EDTA, pH 8.0). Subsequently, we quantified DNA concentration by Quant-iT PicoGreen dsDNA reagent kit (Invitrogen, New York, USA) using a Victor3 Multilabel Counter (Perkin Elmer, Waltham, USA). The purified amplicons were mixed in equimolar concentrations to ensure equal representation of each sample and, were sequenced on an Illumina MiSeq instrument using the V3 reagent kit (2 × 250 cycles).

### Bioinformatics

After demultiplexing raw reads, we visualized the quality profiles of forward and revered reads by uploading the Fastq files into R using package DADA2 (v1.16.0)^35^. We set maximum number of expected errors (E_max) at 0.5 to remove low-quality reads (with expected errors (E) > E_max) from demultiplexed reads. We then remained good-quality regions of reads for further steps by truncation at positions 200 of forward and 140 of reverse reads. Samples with total read counts below 12,000 were not further analyzed. Merged reads were used to generate amplicon sequence variants (ASV) using DADA2 (V1.16.0)^35^. Identified ASVs were aligned to construct a phylogeny with FastTree2^36^. Taxonomy was assigned using the RDP v16 (Ribosomal Database Project—SILVA 1.38 v2) with the set formatted for DADA2 package^37^.

ASVs that were present in less than 5% of all samples as well as reads with a total relative abundance < 0.01% were removed from downstream analysis. At ASV level of taxonomic aggregation, we calculated the alpha-diversity (observed richness and Shannon index) and beta-diversity (Aitchison and Bray–Curtis distances) as measures of within- and between-sample microbial diversity, respectively. To examine the change in alpha-diversity from baseline (M1) to the subsequent time points (M2, M3, M4, M5, M6), we next calculated the delta of the alpha diversity ($d\Delta$) using the formula:

$${\text {delta alpha diversity}} \left( {\text d\Delta } \right) = {\text {alpha diversity of the following, time point  - alpha diversity at M1}} $$

### Statistical analysis

All statistical and computational analysis and visualizations were performed in R v4.1.1 (2021–08-10) using the following packages: reshape2 (v.1.4.4), purrr (v.0.3.4), readr (v.1.3.1), tidyr (v.1.1.2), tibble (v.3.0.4), tidyverse (v.1.3.0)^38^, RColorBrewer (v.1.1–2), ggthemes (v.4.2.0), circlize (v.0.4.10), ComplexHeatmap (v.2.0.0)^39^, ggplot2 (v.3.3.2)^38^, lme4 (v.1.1–27.1), glmmTMB (v.1.1.2.3), ggstatsplot (v.0.9.0)^40^,dplyr (v.1.0.2)^38^, phyloseq (v.1.28.0), microViz (0.7.10.9004)^41^.

Linear mixed effects models were used to test for the effects of antibiotic use, age, and time points on alpha diversity (IBM SPSS Statistics, v 27.0, IBM, Armonk (NY), USA). The model included exposure to antibiotics, age and time point of sample collection as fixed effects and participant ID as a random effect to control for repeated measures. The variance inflation factors (VIF) were calculated prior to model fitting to assess correlated predictor parameters, none were excluded on the basis of collinearity. In addition, the residuals were plotted and checked for homoscedasticity. We next used generalized linear mixed effects models using a negative binomial distribution to test the effect of antibiotic use on the relative abundance of individual bacterial genera (glmmTMB package, R). For each genus, we fitted the model *Genus (Counts)* ~ *Exp_ABx_3codes* + *Age* + *Time_point* + Age *Exp_ABx_3codes + *offset (log (Total_counts))* + *(1|ParticipantID)*. In the model, the variance ‘Exp_ABx_3codes’ is a logical values including three categories that was grouped by number of days since the last antibiotic use prior to sampling as described in the study design.

To examine differentially abundant genus-level taxa between Dutch and Vietnamese individuals, we conducted Analysis of Compositions of Microbiomes with Bias Correction (ANCOM-BC). Differentially abundant genera (*p* < 0.05 upon Bonferroni correction) are presented^42^.

### Ethical approval

The research was approved by the Oxford University Tropical Research Ethics Committee (OxTREC, 49–14), the National Institute of Hygiene and Epidemiology, Vietnam (NIHE) institutional review board and National Hospital for Tropical Diseases, Vietnam.

All methods were performed in accordance with the relevant guidelines and regulations.

We confirm that informed consent was obtained from all participants and/or their legal guardians.

## Results

### Antibiotic use in the Ha Nam cohort (November 2014–June 2015)

To analysis the impact of antibiotic use on the microbiota in the Ha Nam cohort. This observational study investigated gut microbiota responses among 63 individuals belonging to 11 HHs that used at least one course of antibiotics (n = 32) or 11 HHs that did not use antibiotics (n = 31) during the study period. The median age was 28 years (IQR = 8.5–45.2) for those that consumed antibiotics (including 6 subjects aged < 6 years) versus 41 years (IQR = 17.5–52.5) for those who did not (no subjects aged < 6 years).

Among the 32 individuals who used antibiotics during the study period, 20 individuals consumed two or more courses, which resulted in a total consumption of 69 courses of oral antibiotics (Figure S1). First and second generation cephalosporins (n = 52) were most frequently consumed, followed by penicillin (n = 15), trimethoprim (n = 1) and lincosamide antibiotic (n = 1).

The symptoms for which participants reported to have used antibiotics were symptoms of the upper respiratory tract (fever accompanied by cough) for 50 courses, urinary tract combined with upper respiratory tract symptoms for 15 courses and urinary tract combined with stomach complaints for 4 courses. In none of these cases individuals reported to have symptoms of diarrhoea or other intestinal tract symptoms. Therefore, it is unlikely that confounding by indication (i.e., enteric infections rather the antibiotics itself affecting the microbiome) affected our findings.

### Transient effect of antibiotics on microbiota and low diversity at baseline

A total of 373 samples were collected from 63 individuals at month 1 (n = 60), month 2 (n = 62), month 3 (n = 63), month 4 (n = 63), month 5 (n = 63) and month 6 (n = 62) of follow-up.

After filtering of low-quality reads the remaining reads with a median sequencing depth of 81,488 reads per sample (IQR 71,719–93,257) were clustered into 5,367 amplicon sequence variants (ASVs). Upon removal of rare ASVs, defined as ASVs present in less than 5% (n = 19) of the samples and ASVs with an overall abundance of less than 0.01%, a total of 460 ASVs were retained.

**Figure 2:**
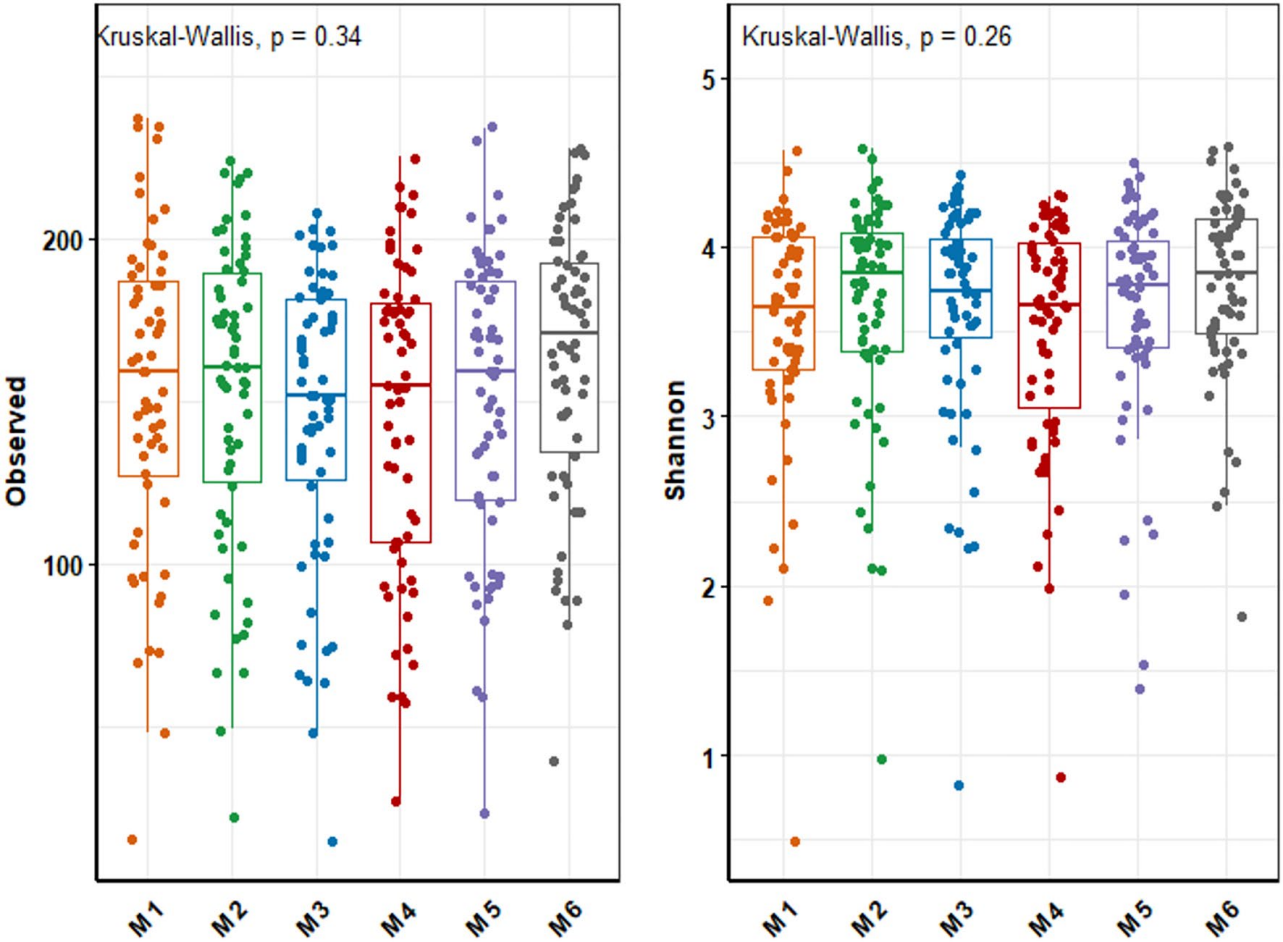
Comparison of alpha diversity (observed richness and Shannon diversity) between follow-up time-points in the study cohort.

**Figure 3:**
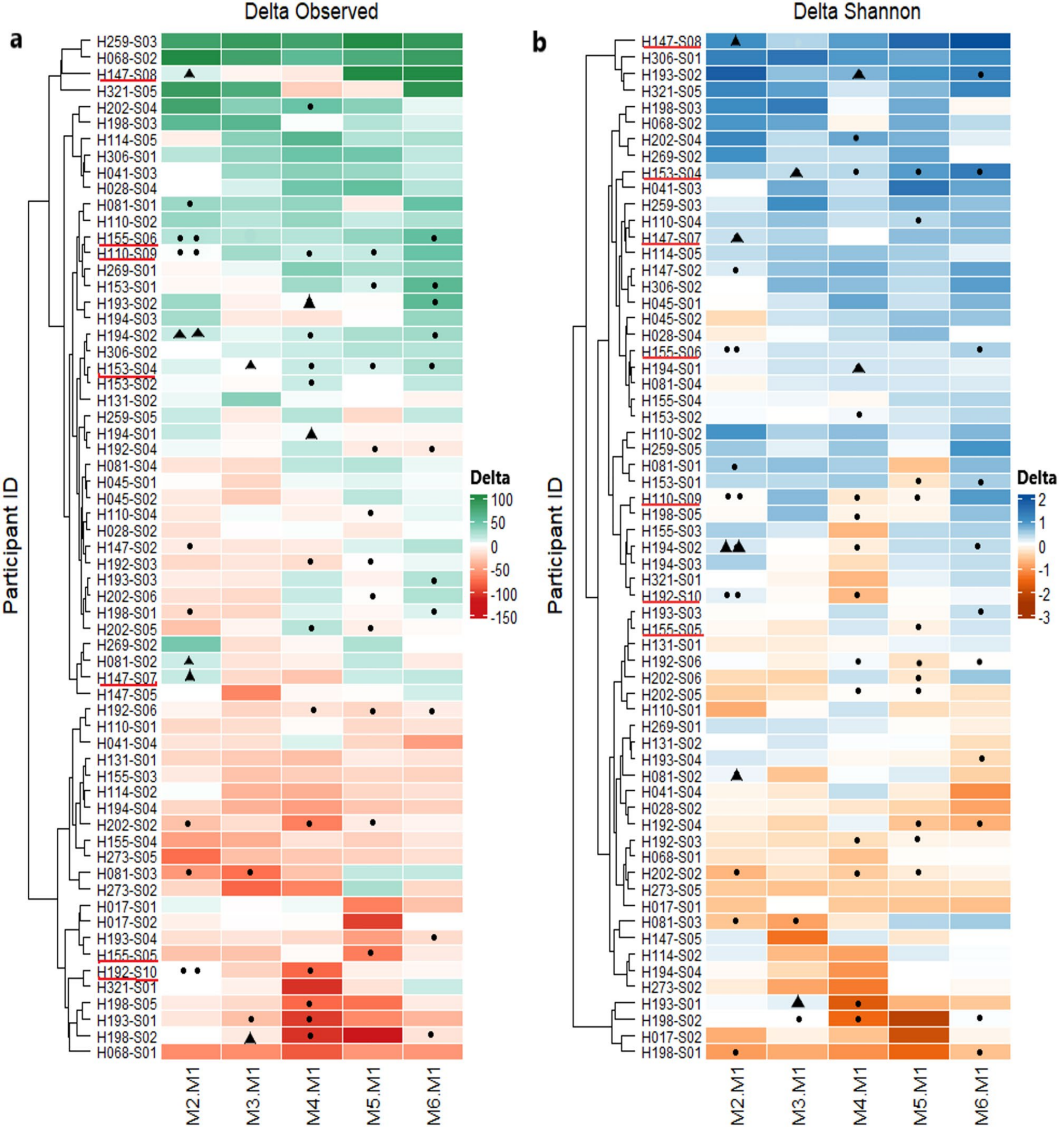
Within-subject shifts in microbial richness and diversity throughout the 6-months follow-up. The heatmaps depict the changes in microbial richness (**a**) and Shannon diversity (**b**) within each study subject between the first month (baseline) and the following months during the study period. Circles and triangles indicate that subjects used antibiotics within four weeks of the later time point (circles for cephalosprorins and triangles for penicillin/amoxicillin). The red lines under the participant ID indicate individuals under 6 years of age. The colour legends (Delta) indicate the difference of alpha diversity from baseline.

We first investigated the richness and diversity of the microbiota. Overall, significant differences during the study period were not observed for both richness and biodiversity (Fig. 2). Although we did not observe a difference in alpha diversity metrics over time at the population-level, we did observe strong perturbations among individuals. At time-points when individuals had consumed antibiotics, the alpha diversity often was reduced as compared to baseline (M1). Differences then gradually narrowed at the subsequent time points, when those individuals did not use antibiotics. The diversity of microbiota from individuals who did not use antibiotics was more stable over 6 months (Fig. 3a, 3b).

To account for the longitudinal design of our study and for potential confounding by age and carriage of extended-spectrum beta lactamases (ESBL) and carbapenemases, we next assessed the effects of antibiotics on the alpha diversity by linear mixed-effects models. Neither the follow-up time-point at which the fecal samples were collected, nor age, significantly affected the observed richness and Shannon index (all p > 0.05) (Table 1). Also, the fecal carriage of enzymes encoding ESBLs (n = 235) and carbapenemases (n = 22) did not affect these alpha diversity indices (Table 1). While antibiotic use was associated with a significant decrease in microbial richness, this effect was only observed when antibiotics were used within two weeks prior to fecal sampling (ß = − 31.3, 95%CI = − 55.3, − 7.3, *p* = 0.011) and had already disappeared when antibiotics were consumed 2–4 weeks prior to fecal sampling (ß = − 1.3, 95%CI = − 34.4, 31.7, *p* = 0.937). Also, the microbial diversity, as measured by the Shannon index, was reduced in samples collected within two weeks after antibiotic use, although this was not statistically significant (ß = − 0.298, 95%CI − 0.686, 0.090, *p* = 0.132). These findings suggest that in the current population the use of antibiotics has only an effect on the microbial richness for a short period, while the microbial diversity is even less affected. In contrast the reduction of microbial diversity remained significantly lower than that in the initial composition after 42 days of treatment with beta–lactam antibiotics, as previously described^11,12^.

**Table 1:** Linear mixed effects models to test for the effects of antibiotic use, age, and time points on alpha diversity. (**a**) The effects of antibiotic use on species richness (Observed), (**b**) on biodiversity (Shannon index).

Variable	Value	b (95% CI)	Standard error b	*p*-value
**a**				
Age (in years)	Age (in years)	0.086 (−0.425, 0.597)	0.256	0.739
Timepoint	months	0.877 (−0.786, 2.539)	0.845	0.3
Antibiotics use	0–2 weeks	−31.344 (−55.372, −7.316)	12.213	0.011
	2–4 weeks	−1.333 (−34.442, 31.775)	16.828	0.937
bla_NDM_ positive	Yes	10.233 (−2.744, 23.211)	6.597	0.122
bla_CTX-M_ positive	Yes	−4.509 (−10.607, 1.590)	3.1	0.147
Timepoint*[Antibiotics use]	Timepoint*[0–2 weeks]	5.641 (0.184, 11.098)	2.774	0.043
	Timepoint*[2–4 weeks]	−2.102 (−11.163, 6.958)	4.605	0.648

b				
Age (in years)	Age (in years)	0.003 (−0.004, 0.009)	0.003	0.448
Timepoint	Months	0.029 (0.002, 0.056)	0.014	0.036
Antibiotics use	0–2 weeks	−0.298 (−0.686, 0.090)	0.197	0.132
	2–4 weeks	0.160 (−0.374, 0.694)	0.271	0.555
bla_NDM_ positive	Yes	0.153 (−0.056, 0.362)	0.106	0.15
bla_CTX-M_ positive	Yes	−0.043 (−0.142, 0.055)	0.05	0.386
Timepoint*[Antibiotics use]	Timepoint*[0–2 weeks]	0.036 (−0.053, 0.124)	0.045	0.428
	Timepoint*[2–4 weeks]	−0.081 (−0.228, 0.065)	0.074	0.274

### Microbiota of Vietnamese as compared to Dutch adults

**Figure 4:**
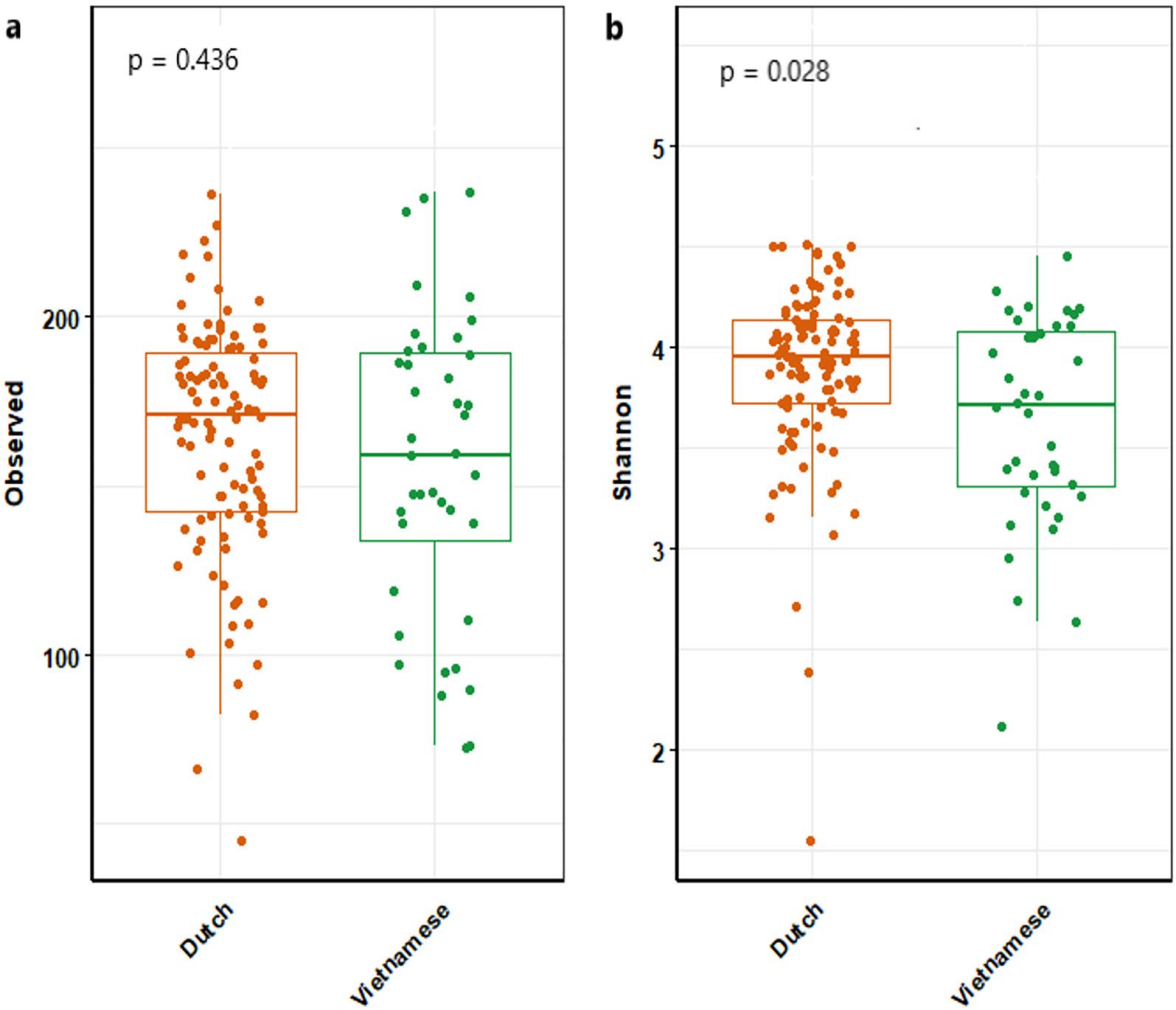
Microbial richness (**a**) and diversity (**b**) in Vietnamese (n = 57) and Dutch healthy adults (n = 106) who did not use antibiotics for at least one year prior to sampling. P-values are based upon linear regression analysis with adjustment for age and sex.

As this modest effect of antibiotic use is in contrast to previous studies^43,44^, we hypothesized that the microbiota diversity of individuals in this cohort might have already been reduced by extensive past antibiotic use to an extent that the effect of further perturbations would be limited. To test this hypothesis, we compared the microbial richness and diversity of the adults in the Ha Nam cohort (n = 63) to that of a cohort of healthy Dutch adults (n = 106). Several previous studies have shown that rural communities in LMICs have a higher microbial richness and diversity compared to individuals in Western countries, mainly as a result of a diet that is rich in fibers and low in animal fat and protein. Indeed, most Vietnamese adults take grains (96.4%) and vegetables (93.9%) every day^45^. Meanwhile, less than 10% of the Dutch take the recommended amount of food originate from plant^46^. Moreover, on average, each Vietnamese person consumes 134 g meat per day^47^, much lower than the Dutch’s (450 g meat and dairy products, daily)^46^. However, the microbiota of the Ha Nam cohort participants showed a statistically significantly lower microbial diversity at baseline with Shannon median 3.69 (IQR 3.31–4.11) when compared to the Dutch individuals with median 3.95 (IQR 3.72–4.13), (*p* = 0.028). This supports the hypothesis of a reduction in microbial diversity as a consequence of frequent antibiotic exposure (Fig. 4).

**Figure 5:**
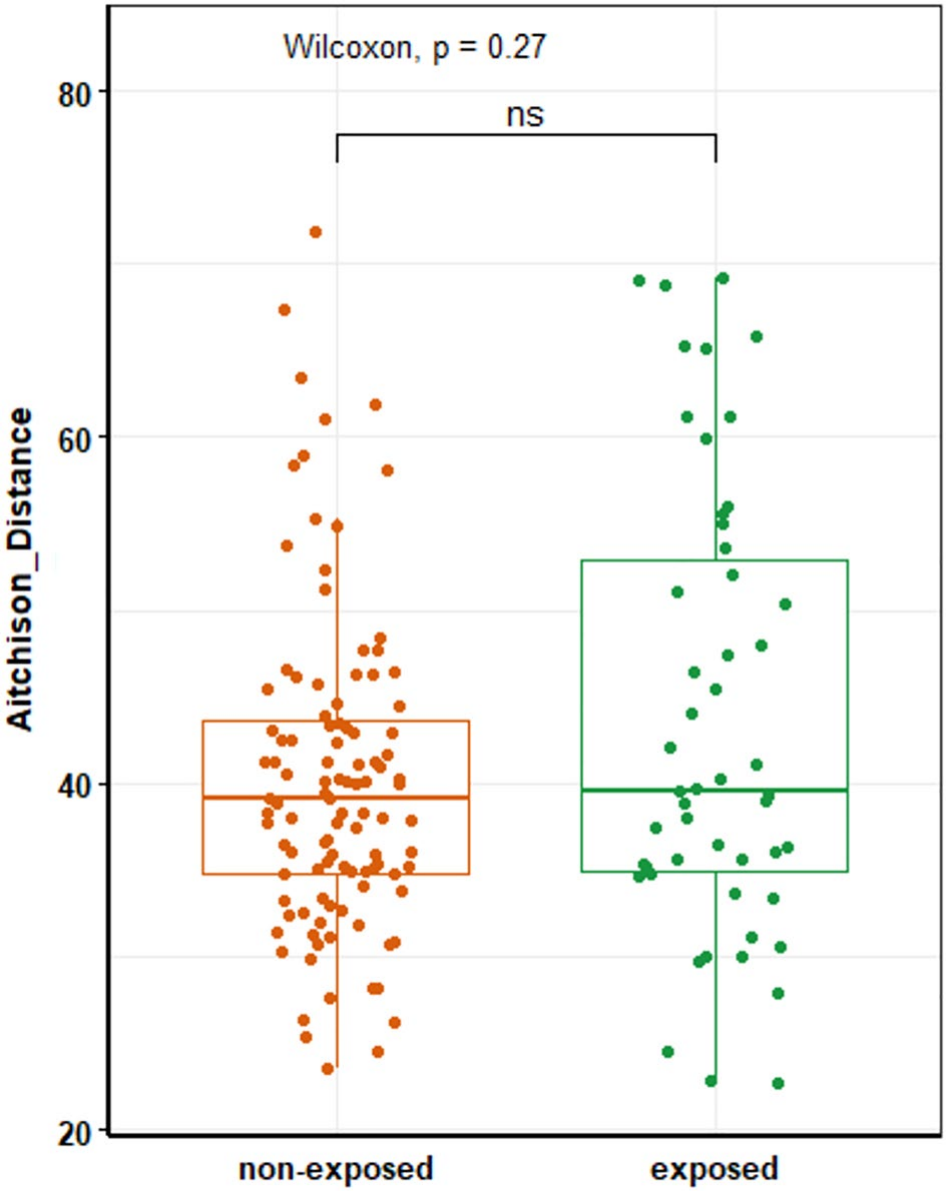
Comparison of within-subject microbiota dissimilarity (Aitchison distance) between samples at the adjacent time point before and after exposure to antibiotics (exposed) versus within-subject microbiota dissimilarity between adjacent samples without intermittent antibiotic exposure.

We next examined whether the microbial resistance to antibiotic-induced pulse perturbations among participants in the Ha Nam cohort was associated with the similarity of their microbiota to that of Dutch individuals. Ordination analyses showed that participants with a more resistant microbiota did neither cluster apart from individuals with a less resistant microbiota nor was the microbiota of these participants more (dis)similar to that of Dutch individuals (Figs. 5, S3 and S4).

Analysis of the differentially abundant taxa at genus level showed that many bacterial genera, including *Prevotella, Blautia,* and *Holdemanella*, of which specific species/strains have previously been identified as Volatile and/or Associated Negatively with Industrialized Societies of Humans (VANISH)^48^, were depleted in Dutch as compared to Vietnamese individuals. Next to these vanishing microbes, also members of the *Enterobacteriaceae* family including, *Klebsiella* and *Escherichia-Shigella* were enriched in the Vietnamese individuals. On the other hand, *Akkermansia* and *Alistipes*, genera of which members have been classified as BloSSUM (Bloom or Selected in Societies with Urbanization/Modernization) microbial taxa, were enriched in the Dutch individuals (Figure S5).

### Impact of antibiotic use on microbial community structure and composition

To examine the impact of antibiotic use on the microbial community structure, we calculated the dissimilarity between samples using the Aitchison distance metric. As expected, Aitchison distances between samples from the same individuals were significantly lower than the distance between samples from different individuals (*p* < 0.001, Wilcoxon test) (Supplementary Figure S2). However, Principal Component Analyses (PCA) did neither reveal separation of samples into distinct microbiota-based clusters by time points nor by antibiotics use (Supplementary Fig. S6).

**Figure 6:**
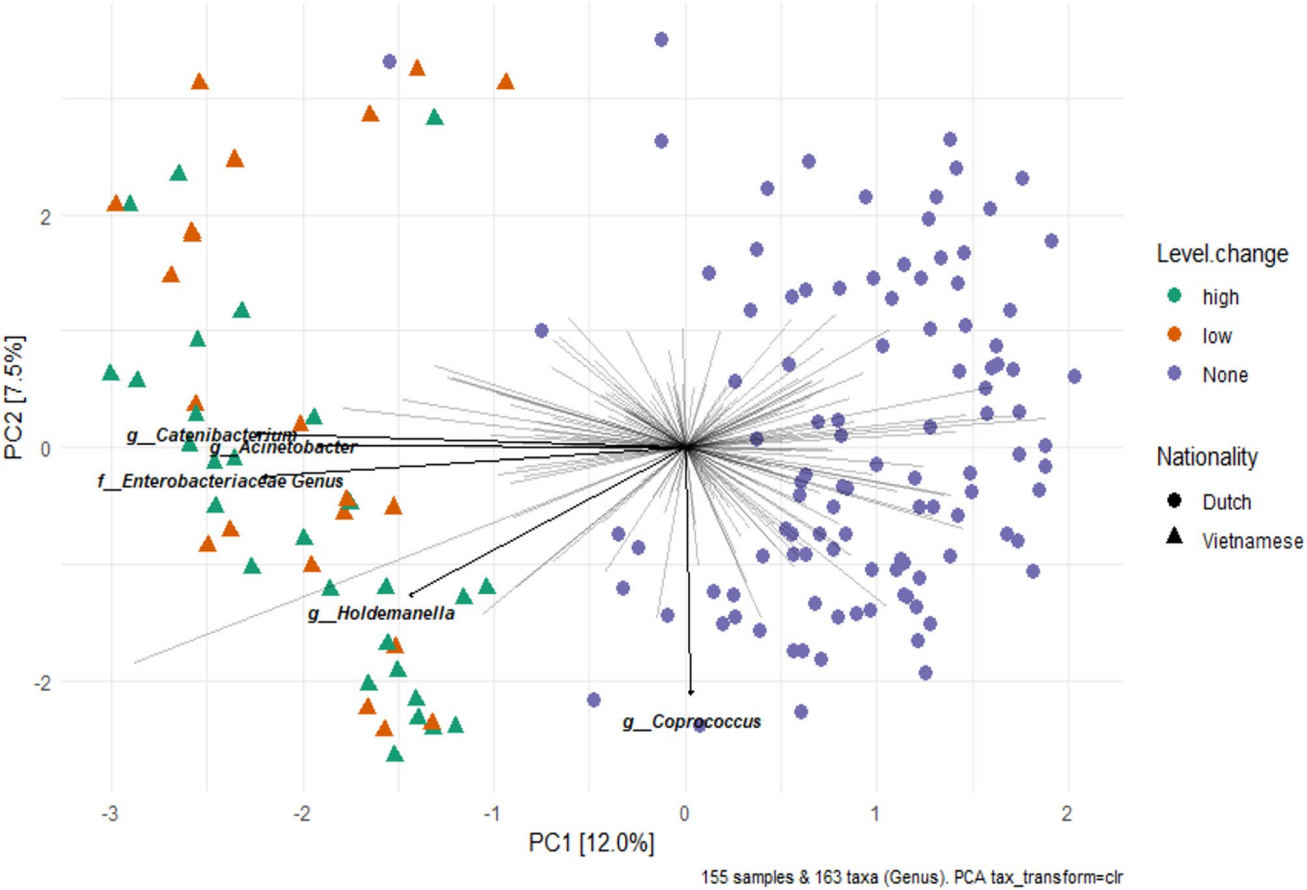
Principal Component Analyses (PCA) comparing the resistance to change of the microbiota of Vietnamese subjects who used antibiotics (both adults and infants, n = 39) to the microbiota of Dutch individuals (n = 106) without antibiotic exposure. Samples of Vietnamese subjects were collected in the month preceding the antibiotic use and are coloured according to the resistance to antibiotics defined as the Aitchison distance between the depicted pre-antibiotic sample and the sample collected after antibiotic use (not depicted in this plot), with a large dissimilarity (within-subject Aitchison distance above the median) depicted as green triangles and low dissimilarity as orange triangles.

We examined the impact of antibiotic use on the stability of the microbial community structure among subjects who used antibiotics at any given time during the study period. To this end, we compared the within-subject Aitchison distance of samples collected at the adjacent time points before and immediately after exposure to antibiotics to the within-subject Aitchison distance of the same individuals but between adjacent samples without intermediate exposure to antibiotics. These analyses revealed that the stability in microbial community structure was not affected by antibiotic use (Fig. 6). Visualization of the longitudinal changes in the microbial community structure of a random selection (for visual purposes) of participants that did or did not use antibiotics also corroborated the findings that the stability in the microbial community structure was not affected by antibiotic use (Fig. S7).

We next examined whether exposure to antibiotics was associated with changes in the relative abundance of bacterial genera by generalized linear mixed effects models using a negative binomial distribution. We used exposure to antibiotics, age and time-point as fixed effects, and individual subjects as random effect to control for repeated measures. None of the genera were statistically significantly altered upon antibiotic use upon correction for multiple comparisons (FDR > 0.05) (see Supplementary—Table S1). This result suggested that the abundance of taxa was not disturbed, including those of bacteria susceptible to cephalosporins, even immediately after exposure to antibiotics.

Lastly, the linear mixed effects models analysis showed that the number of ARGs in faecal sample collected before antibiotic use was not associated with the subsequent shifts in observed richness and Shannon diversity upon antibiotic exposure (all *p*-value > 0.05, Supplementary, Tables S2 and Table S3). Also, ordination analysis did not show any correlation between the overall microbial community structure and the number of ARGs in the faecal samples (Fig. S8).

## Discussion

This study examined the effect of commonly used antibiotics on gut microbiota among residents of a rural community with frequent antibiotics consumption and high levels of antibiotic resistance^24,30,49^. We observed modest reduction of gut microbial diversity within less than 31 days of antibiotic consumption, contrasting with a more pronounced reduction in cohorts treated by cephalosporins^50^ or other antibiotics^12,51^. Detailed analysis of microbial diversity showed that exposure to common antibiotics only resulted in the reduction of microbial richness but did not affect the biodiversity of microbiota in general, in contrast to observations in other cohorts^17,44^. These results highlight less diversity in microbiota at baseline as compared to Dutch individuals, from a setting with less antibiotic use.

Previously a strong link between the baseline microbiota and alterations in the resistome upon antibiotic treatment^52^. On the other hand, it is conceivable that the baseline resistome would also impact the shifts in microbiota composition upon antibiotic exposure since antibiotics do not eliminate antibiotic resistant bacteria in the microbiota^6^. In our previous study, we showed the extreme abundance of ARGs conferring resistance to beta lactam antibiotics in this community. Likely due to this high resistance background, we did not observe an alteration of *Klebsiella.spp* and other *Enterobacterales* after a course of antibiotics, in contrast with a previous report conducted in a healthy Caucasian population using one course of broad-spectrum antibiotics^12^.

Perhaps, repeated exposure to cephalosporin antibiotics over a short period has resulted in the microbiota reaching a state of tolerance to the antibiotic treatments. Consequently, the further antibiotic perturbations, particularly on the microbiota of our study cohort with a high level of resistance genes, would be transient, and rapidly return to their prior state.

However in contrast to this hypothesis, the microbiota of individuals with no or low number of ARGs against beta-lactam antibiotics and colistin was not more strongly perturbated by antibiotic exposure than the microbiota of individuals containing a high number of ARGs. It should however be noted that there are many more beta-lactamases, including penicillinases and cephalosporinases than the extended-spectrum beta-lactamases and carbapenemases quantified in our study. It is therefore possible that the classification of low and high ARGs does not adequately reflect the true diversity of beta-lactamases in the respective fecal samples. Future studies using shotgun metagenomics should be conducted to disentangle this bi-directional impact of the microbiota and its resistome under antibiotic exposure in more detail.

Low vaccination coverage, coupled with poor water, sanitation, and hygiene (WASH) infrastructure, residents vulnerable to infection and dependent on antibiotics for treatment^17^, have been recognized as major factors driving antibiotic consumption. Indeed, a research conducted in Vietnam and other LMICs, has shown proportion of households reporting use of antibiotics in the previous month in the household survey was 45% (416/925)^49^. Possibly, individuals in the control group have used antibiotics without being aware or recording, leading to an alternative microbiota composition with a similar richness and diversity to their prior composition. Consequently, it is difficult to observe the difference in richness and biodiversity between them and antibiotics users. In addition, people of rural communities of LMICs like Vietnam, normally have a diet with higher content than western diets, while studies having shown fiber rich diets were associated with higher diversity of microbiota^53,54^. When comparing the microbiota of participants of the Ha Nam cohort to that the microbiota of Dutch individuals, we observed differences in the abundance of specific microbial genera in line with previous studies comparing traditional and industrialized populations^48^. However, in contrast, our results showed a significantly lower diversity in the microbiota of the participants of the Ha Nam cohort as compared to those from a Dutch cohort with less antibiotic use, supporting our hypothesis that Ha Nam cohort participants have chronically disrupted microbiota, with a lower diversity, due to frequent antibiotic exposure.

The study has several limitations. The objective, to evaluate the effect of antibiotic use on microbiota in a community, may require a longer follow-up study, and the sample size was constrained by monthly sample collection. Currently, the definition of a healthy microbiome has not been established. Therefore, we could not conclude with certainty that the gut microbiota of this cohort is in an unhealthy steady state. Moreover, recorded antibiotic use in our community was predominantly with earlier generation cephalosporins (access group), which are less potent compared to later generation (watch group) or other restricted antibiotic (reverse group) classes^55^. This contrasts with another recent study, where the use of both access- (59.0%) and watch (39.3%) group antibiotics was commonly observed in rural Vietnam^56^.This suggest that it needs an investigation the response of the human gut microbiota to the new patterns of antibiotic use. Nonetheless, the results presented here support the hypothesis that frequent or chronic antibiotic exposure may push the microbiota to a different steady state that is less diverse but more resilient to disruption by antibiotic use. Further research including comparisons between populations with different exposures is needed to provide more evidence for this hypothesis. Furthermore, the potential health consequences of this altered microbiota composition should be considered.

## Conclusions

This study has added a line to the long-term impacts of antibiotic use that, with frequent exposure to antibiotic, the gut microbiota adapts to a state that is little influenced by antibiotics of longitudinal study targeting long term influence of antibiotics on microbiota in similar settings.

## Supplementary Information

Supplementary Information 1.

Supplementary Information 2.

## Data Availability

The data for this study have been deposited in the European Nucleotide Archive (ENA) at EMBL-EBI under accession number PRJEB53339 (https://www.ebi.ac.uk/ena/browser/view/ PRJEB53339). Accession Numbers have been listed in Supplementary (Table S5: Accession Number).

## Abbreviations

LMICs: Lower and middle income countries
PCA: Principal component analyses
ARGs: Antibiotic resistance genes
AMR: Antimicrobial resistance
DNA: Acid deoxyribonucleic
