# Supplementary material for: Moderate and transient impact of antibiotic use on the gut microbiota in a rural Vietnamese cohort
Source: Sci Rep. 2022 Nov 23;12:20189. doi: 10.1038/s41598-022-24488-9 (PMC9691687; doi:10.1038/s41598-022-24488-9)
Supplement: Supplementary file 1 — Supplementary Information 1. [file 41598_2022_24488_MOESM1_ESM.pdf]

Supplementary Figures

Moderate and transient impact of antibiotic use on the gut microbiota in a rural Vietnamese cohort.

Authors: Vu Thi Ngoc Bich, Ngoc Giang Le, David Barnett, Jiyang Chan, Niels van Best, Tran Dac Tien, Nguyen Thi Hien Anh, Tran Huy Hoang, H. Rogier van Doorn, Heiman F.L Wertheim, and John Penders

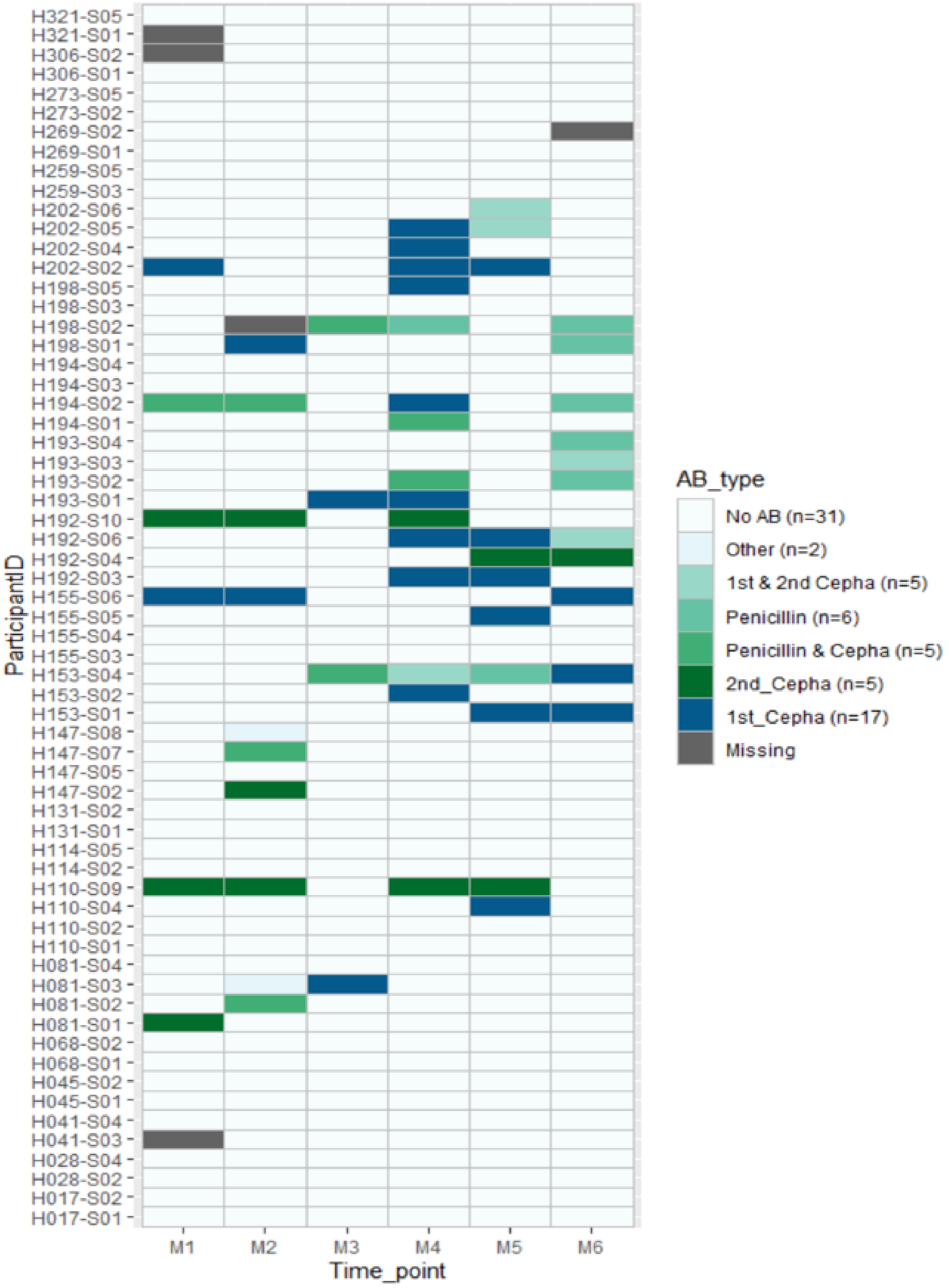

Figure S1: Antibiotic use of individuals in 6 months of study period.

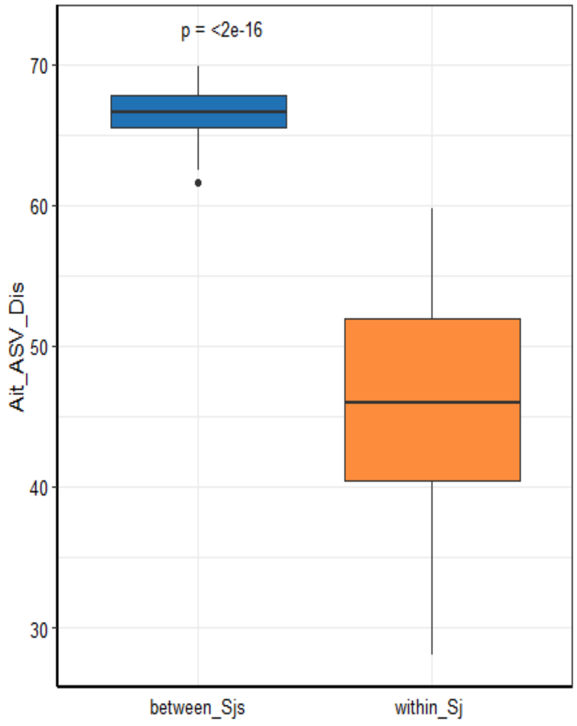

Figure S2: Within-subject (within_Sj) versus between-subject (between_Sjs) microbiota dissimilarity as indicated by the Aitchison distance.

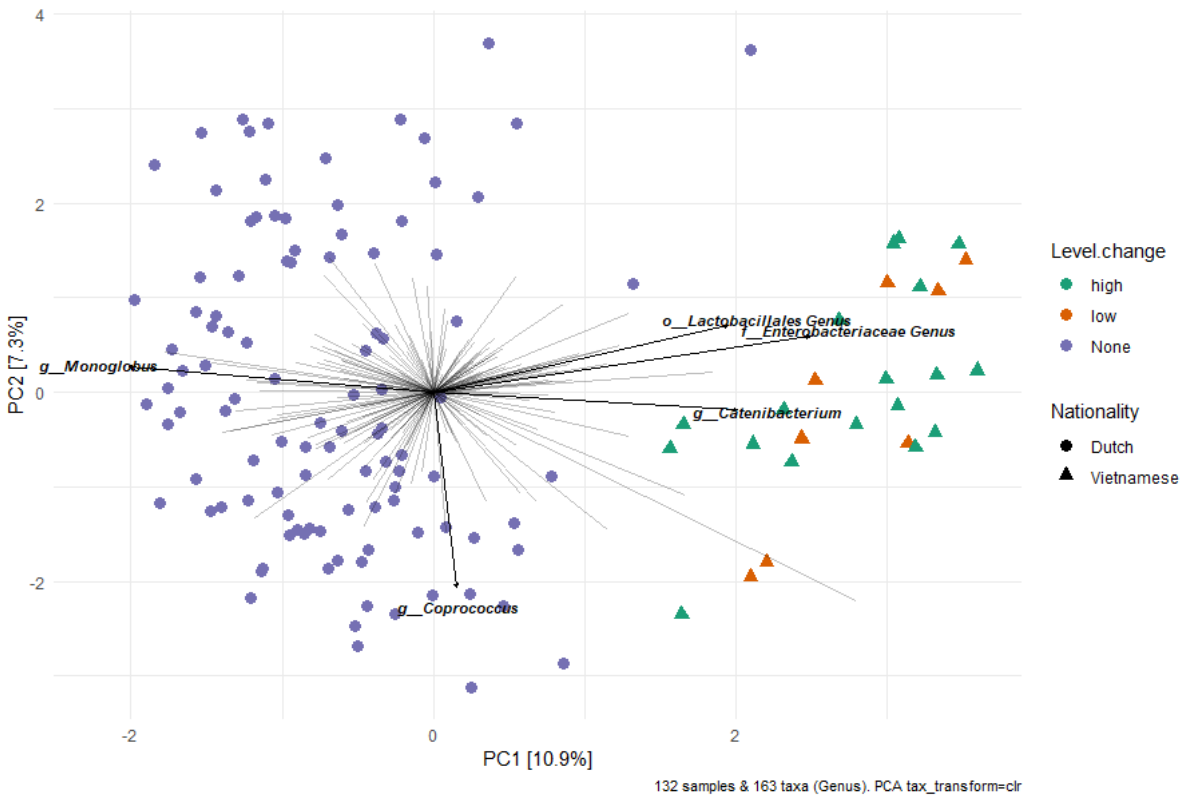

Figure S3: Principal Component Analyses (PCA) comparing the resistance to change of the microbiota of Vietnamese adults who used antibiotics (n = 26) to the microbiota of Dutch adults (n=106) without antibiotic exposure. Samples of Vietnamese subjects were collected in the month preceding the antibiotic use and are coloured according to the resistance to antibiotics defined as the Aitchison distance between the depicted pre-antibiotic sample and the sample collected after antibiotic use (not depicted in this plot), with a large dissimilarity (within-subject Aitchison distance above the median) depicted as green triangles and low dissimilarity as orange triangles**.**

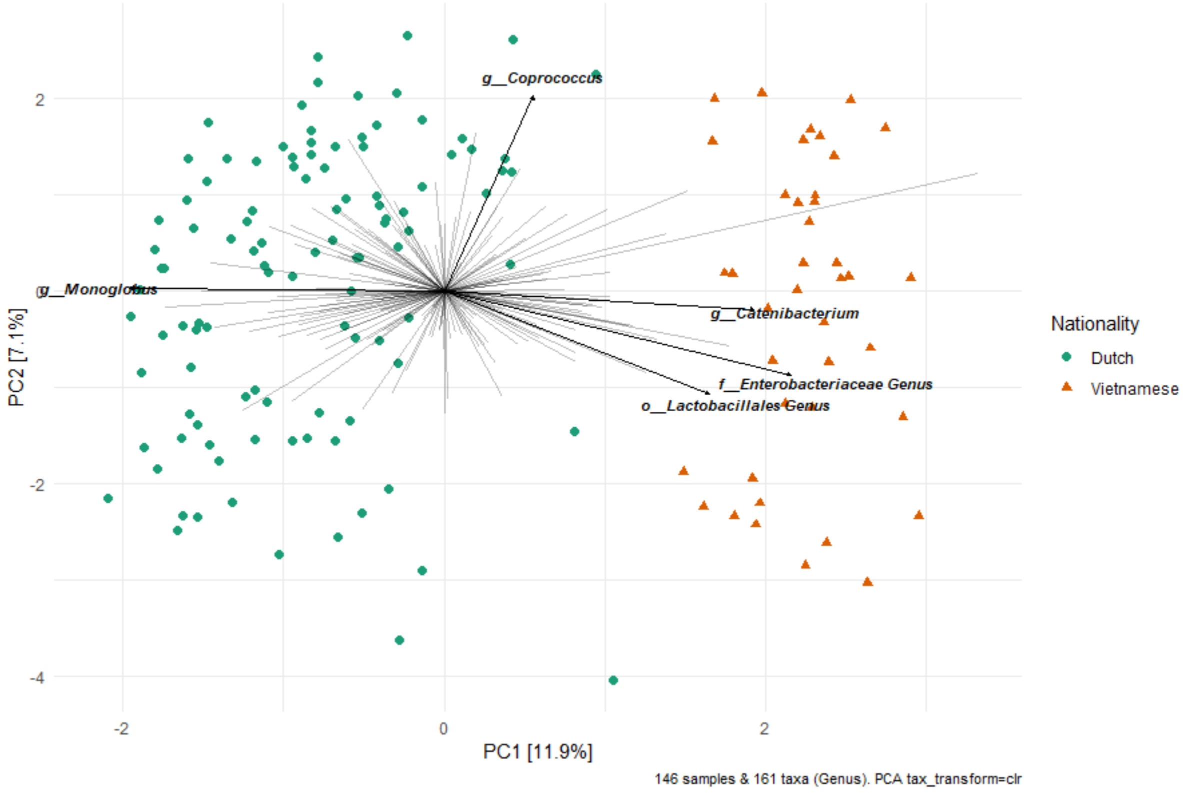

Figure S4: Principal Component Analyses (PCA) comparing the baseline microbiota of Vietnamese adults (n = 40) to the microbiota of Dutch individuals (n=106). Vectors depict the microbial genera with the strongest influence on the ordination of samples along the first two components.

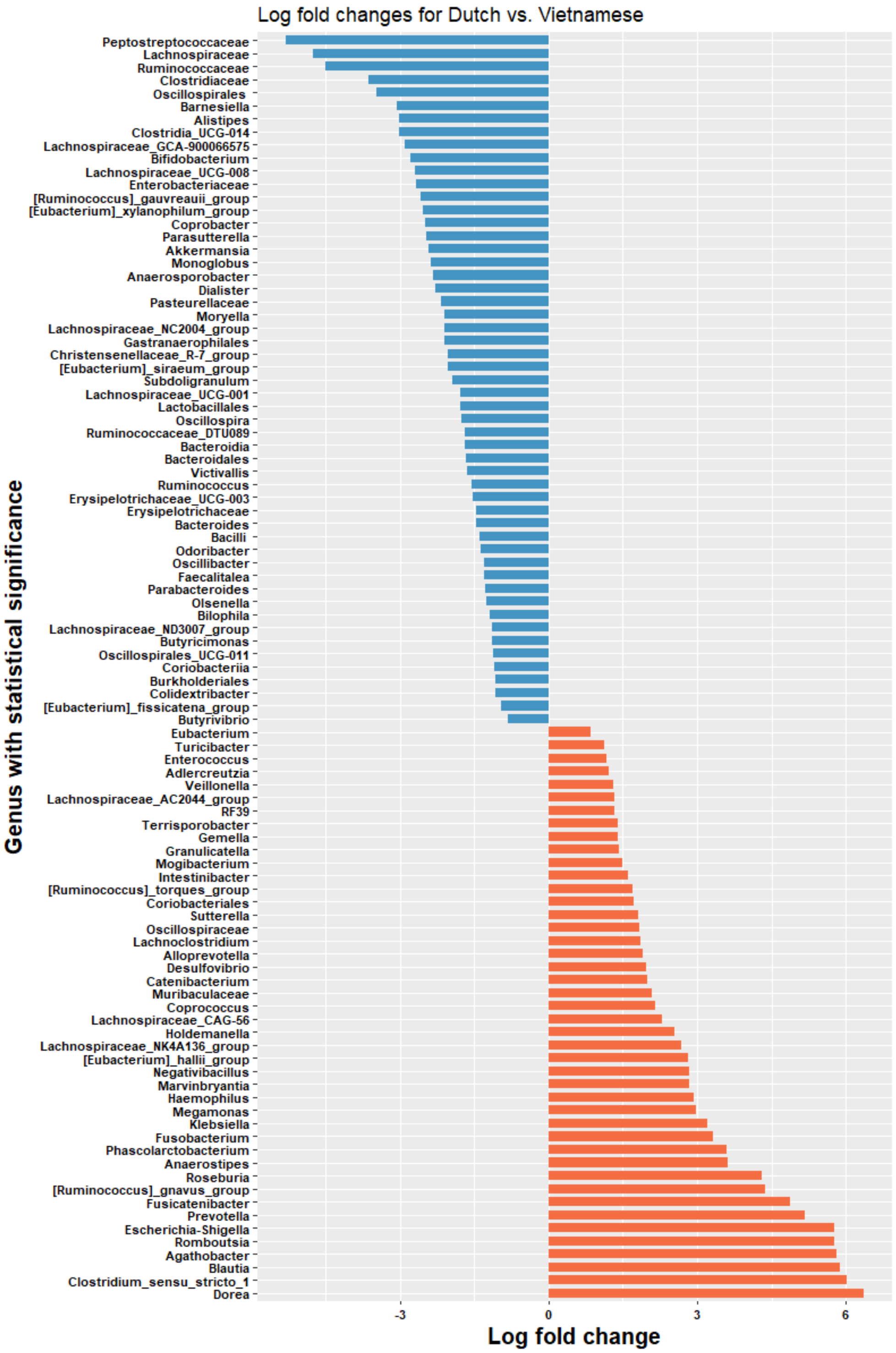

Figure S5: Differential abundant microbial genera between Dutch and Vietnamese cohort. Data are represented by effect size (log fold change) derived from the ANCOM-BC model. Only genera with $p < 0.05$ upon Bonferroni adjustment are depicted. Exact adjusted p values can be found in Supplementary (Table S4: ANCOM-BC).

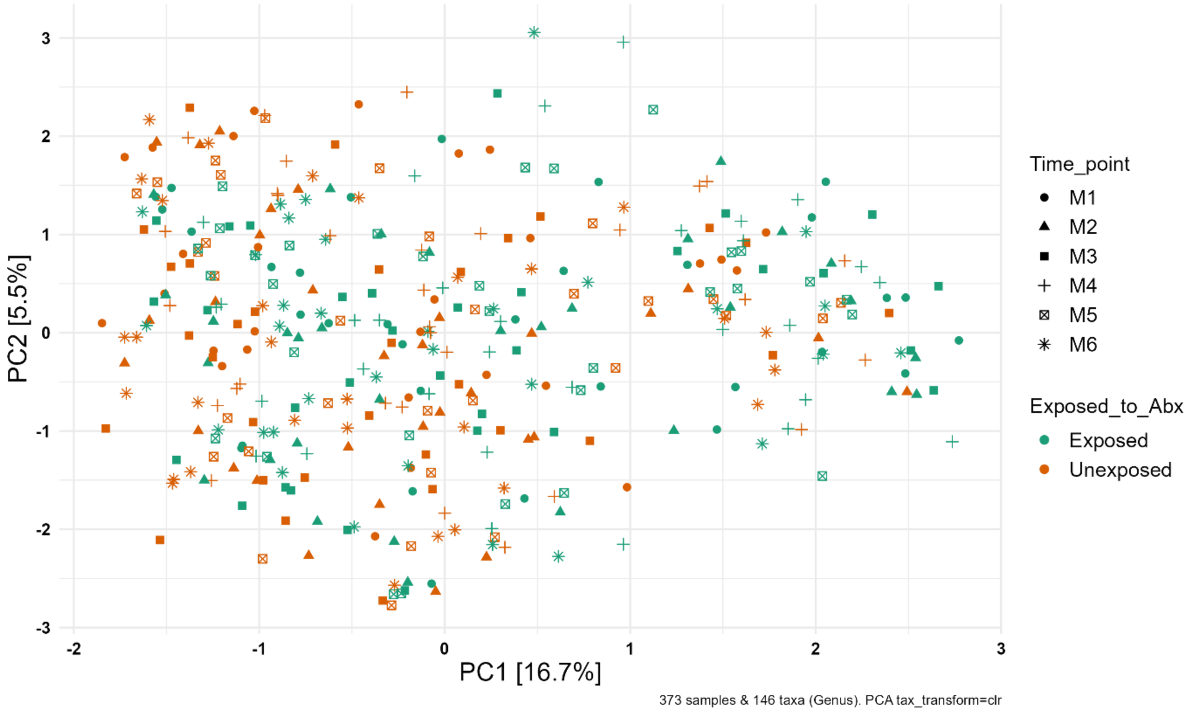

Figure S6: Principal Component Analysis (PCA) (at genus level of taxonomic aggregation) by time points indicated by M1, M2, M3, M4, M5, M6 and by antibiotics use (green indicates samples from individuals who did not used ABs, orange indicates samples from antibiotic users). Exposed and unexposed groups indicate individuals who used and who did not use antibiotics during 6 month of the study period, respectively.

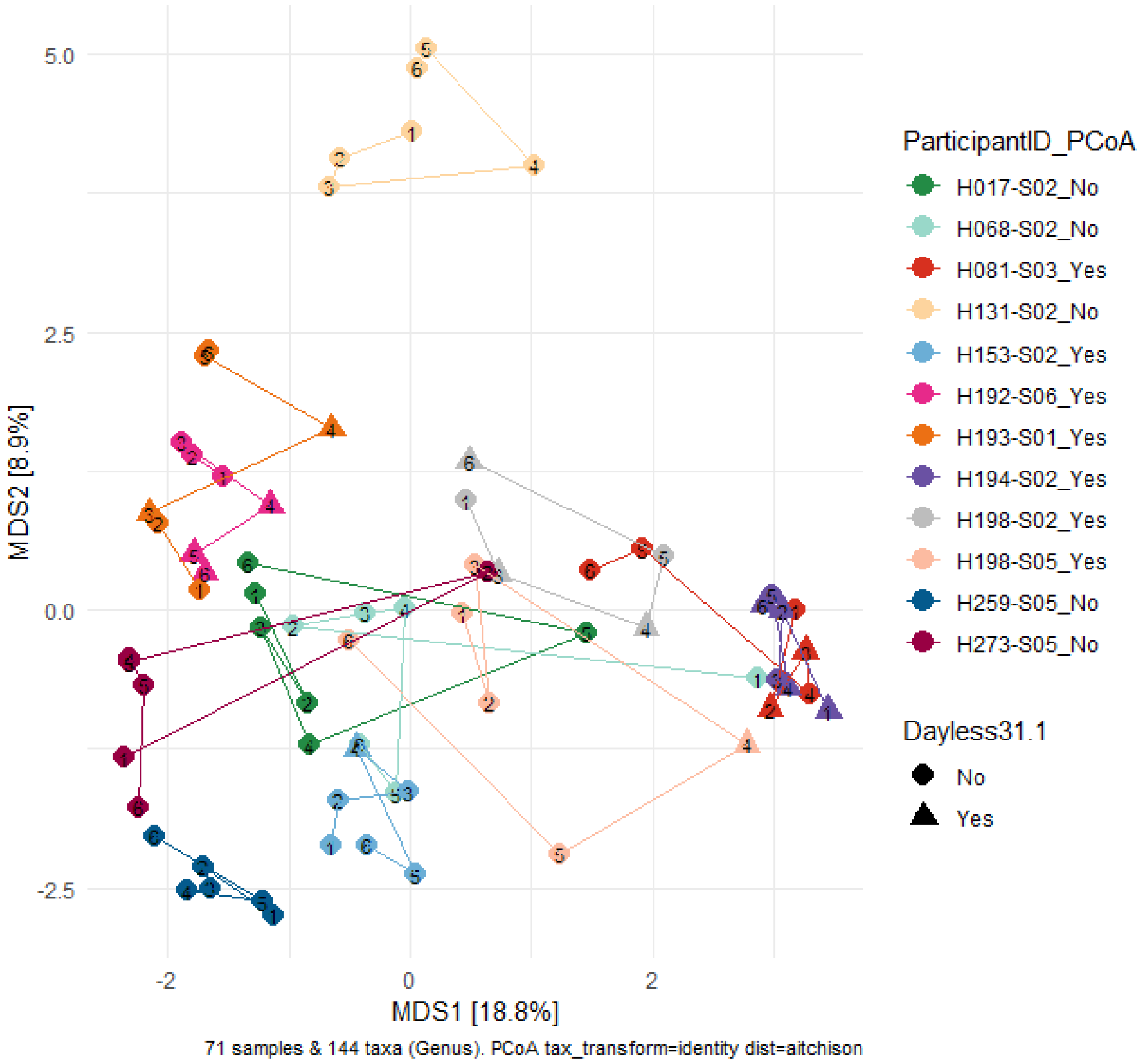

Figure S7: PCoA (at genus level of taxonomic aggregation) visualizing the trajectories in microbiota shifts (Aitchison distance) of a random selection of subjects who used antibiotics (Yes, n=7) and individuals who did not use antibiotics (No, n=5).

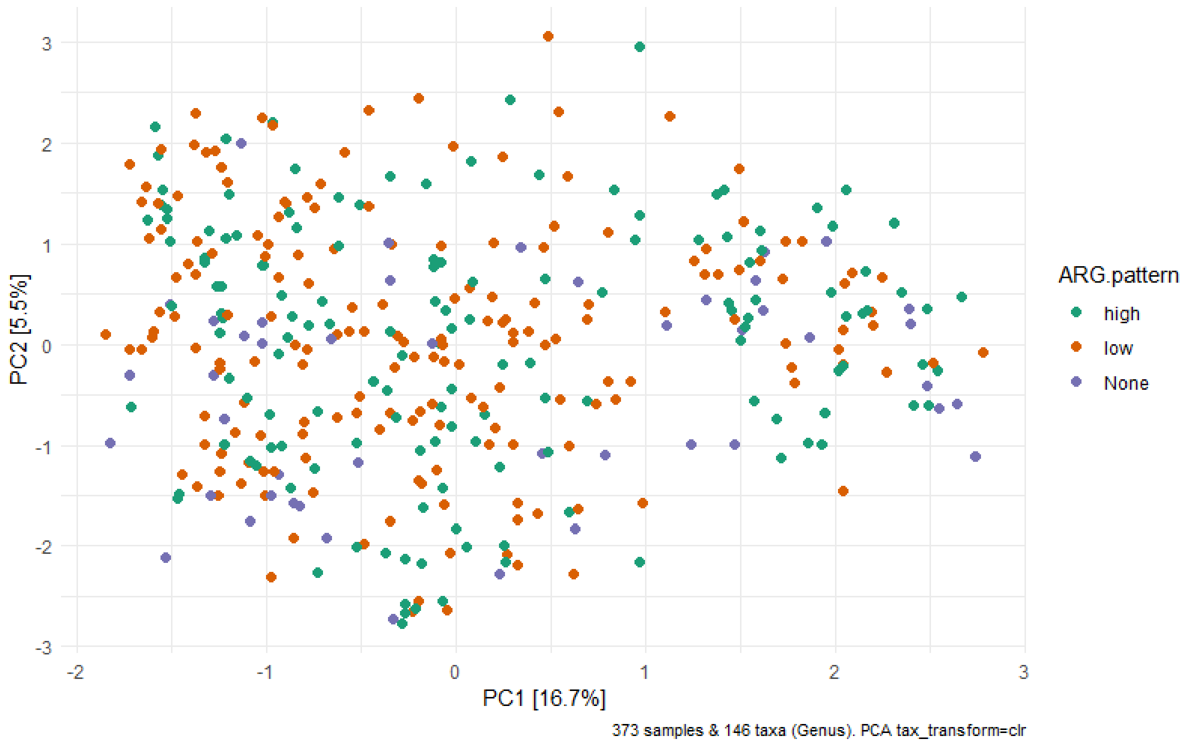

Figure S8: Principal Component Analyses PCA (at genus level of taxonomic aggregation) by ARGs profiles (green indicates samples with >= 3 ARGs (high) encoding extended-betalactamases (CTX-M-1, CTX-M-2, CTX-M-9), carbapenemases (NDM) and plasmid-mediated colistin resistance (MCR-1, MCR-3), orange indicates samples with <3 ARGs (low) and purple indicates samples negative (none) for the above-mentioned ARGs).
